# A Novel Four-Way Complex Variant Translocation Involving Chromosome 46,XY,t(4;9;19;22)(q25:q34;p13.3;q11.2) in a Chronic Myeloid Leukemia Patient

**DOI:** 10.3389/fonc.2016.00124

**Published:** 2016-05-30

**Authors:** Muhammad Asif, Mohammad Sarwar Jamal, Abdul Rehman Khan, Muhammad Imran Naseer, Abrar Hussain, Hani Choudhry, Arif Malik, Shahida Aziz Khan, Maged Mostafa Mahmoud, Ashraf Ali, Saima Iram, Kashif Kamran, Asim Iqbal, Zainularifeen Abduljaleel, Peter Natesan Pushparaj, Mahmood Rasool

**Affiliations:** ^1^Department of Biotechnology, Balochistan University of Information Technology, Engineering and Management Sciences (BUITEMS), Quetta, Pakistan; ^2^Office of Research Innovation and Commercialization, Balochistan University of Information Technology, Engineering and Management Sciences (BUITEMS), Quetta, Pakistan; ^3^King Fahd Medical Research Center (KFMRC), King Abdulaziz University, Jeddah, Saudi Arabia; ^4^Obesity and Diabetes Research Laboratory, Department of Chemistry, University of Azad Jammu and Kashmir, Muzaffarabad, Pakistan; ^5^Center of Excellence in Genomic Medicine Research (CEGMR), King Abdulaziz University, Jeddah, Saudi Arabia; ^6^Department of Biochemistry, Faculty of Science, Center of Innovation in Personalized Medicine, King Fahd Center for Medical Research, King Abdulaziz University, Jeddah, Saudi Arabia; ^7^Institute of Molecular Biology and Biotechnology (IMBB), The University of Lahore, Lahore, Pakistan; ^8^Department of Molecular Genetics and Enzymology, Division of Human Genetics and Genome Research, National Research Centre, Giza, Egypt (Affiliation ID 60014618); ^9^Bolan Medical Hospital, Quetta, Balochistan, Pakistan; ^10^Faculty of Life Sciences, University of Balochistan, Quetta, Pakistan; ^11^Department of Medical Genetics, College of Medicine, Umm Al-Qura University, Makkah, Saudi Arabia

**Keywords:** Philadelphia chromosome, complex variant translocation, BCR–ABL gene, chronic myeloid leukemia

## Abstract

Philadelphia (Ph) chromosome (9;22)(q34;q11) is well established in more than 90% of chronic myeloid leukemia (CML) patients, and the remaining 5–8% of CML patients show variant and complex translocations, with the involvement of third, fourth, or fifth chromosome other than 9;22. However, in very rare cases, the fourth chromosome is involved. Here, we found a novel case of four-way Ph+ chromosome translocation involving 46,XY,t(4;9;19;22)(q25:q34;p13.3;q11.2) with CML in the chronic phase. Complete blood cell count of the CML patient was carried out to obtain total leukocytes count, hemoglobin, and platelets. Fluorescence *in situ* hybridization technique was used for the identification of BCR–ABL fusion gene, and cytogenetic test for the confirmation of Ph (9;22)(q34;q11) and the mechanism of variant translocation in the bone marrow. The patient is successfully treated with a dose of 400 mg/day imatinib mesylate (Gleevec). We observed a significant decrease in white blood cell count of 11.7 × 10^9^/L after 48-month follow-up. Patient started feeling better generally. There was a reduction in the swelling of the body, fatigue, and anxiety.

## Introduction

Chronic myeloid leukemia (CML) is triggered due to the t(9;22)(q34;q11) translocation between the long arms of chromosomes 9 and 22, called as the Philadelphia (Ph) chromosome (1). In these patients, bone marrow myeloid hyperplasia, an elevated myeloid and erythroid cells, and platelets in the peripheral blood were observed (2). This translocation was identified in more than 90% of the CML patients (3, 4), and the variant/complex translocation was observed in 5–8% of cases with an involvement of additional third, fourth, or fifth chromosome (5–7).

The imatinib mesylate is commonly used as the first-line oral treatment of CML patients (6). It blocks the BCR–ABL tyrosine kinase activity and subsequently induces apoptosis followed by the reduction in the proliferation of BCR–ABL-expressing cells in both CML and acute lymphocytic leukemia (ALL). The treatment of CML patients with imatinib significantly increased the survival and improved the quality of life (8).

Potentially imatinib meysylate inhibits the BCR/ABL and platelet-derived growth factor receptor (PDGFR) tyrosine kinase activities. This deactivates downstream signaling by reducing cell proliferation and augmenting apoptosis. Imatinib mesylate (Gleevec) therapy has significantly increased the efficacy in 0–34% of Ph-positive cells with t(9;22) translocation along with other complex translocations causing BCR/ABL gene fusion and subsequent clonal evolution (9, 10). In this study, for the first time, we present a four-way Ph translocation 46,XY,t(4;9;19;22)(q25:q34;p13.3;q11.2) in a CML patient with a new complex rearrangement between chromosomes 4 and 19 as well as 9 and 22.

### Report

A 45-year-old male patient was diagnosed with CML on 19 October, 2012. The hematological parameters were hemoglobin (Hb) 10.0 g/dL (normal range, 14–18 mg/dL), MCV 59.6 fL (normal range, 76–95 fL), MCH 19 pg (normal range, 27–32 pg), MCHC 31.9% (normal range, 30–35), hematocrit 31.4% (normal range, 40–54%), red blood cell (RBC) 5.26 × 10^9^/L (normal range, 4.5–6.5 × 10^9^/L), white blood cell (WBC) 160.7 × 10^9^/L (normal range, 4–11 × 10^3^/μL × 10^9^/L), neutrophils 58% (normal range, 40–75%), lymphocytes 2% (normal range, 20–45), eosinophils 3% (normal range, 1–6%), monocytes 4% (normal range, 2–10%), basophils 4% (normal range, 0–1%), metamyelocytes 15% (normal range, 0–0%), myelocytes 15% (normal range, 0–0%), blast cells 2% (normal range, 0–0%), and platelets 223 × 10^9^/L (normal range, 150–400 × 10^9^/L). Peripheral film showed dimorphic picture, anisocytosis, hypochromic, microcytic, polychromasia, polychromasia, tear drop cells, and nucleated RBC. A written informed ethical consent was taken from the patient before the study according to Helsinki declaration. The ethical committee of Balochistan University of Information Technology, Engineering and Management Sciences (BUITEMS), Quetta, Pakistan, had given the approval for this study.

## Materials and Methods

### Complete Blood Count Laboratory Test

Complete blood count (CBC) was performed to measure the levels of WBCs, RBCs, Hb, and platelets in the CML patients. CBC was performed using an Automatic Hematological Analyzer (Nihon Khoden, Japan) within 2 h of blood sampling. An increase in WBCs and lower levels of RBCs and platelets confirmed the leukemia, and these patients were considered for further examination.

### Cytogenetic Analysis

Chromosome analysis using GTG banding was done as described previously (9). Karyotyping was performed in 25 metaphases from unstimulated bone marrow samples according to the nomenclature of the International System for Human Cytogenetics (11).

### Fluorescence *In Situ* Hybridization

Fluorescence *in situ* hybridization was performed to detect BCR/ABL as described previously (12).

## Results

**Figure 1:**
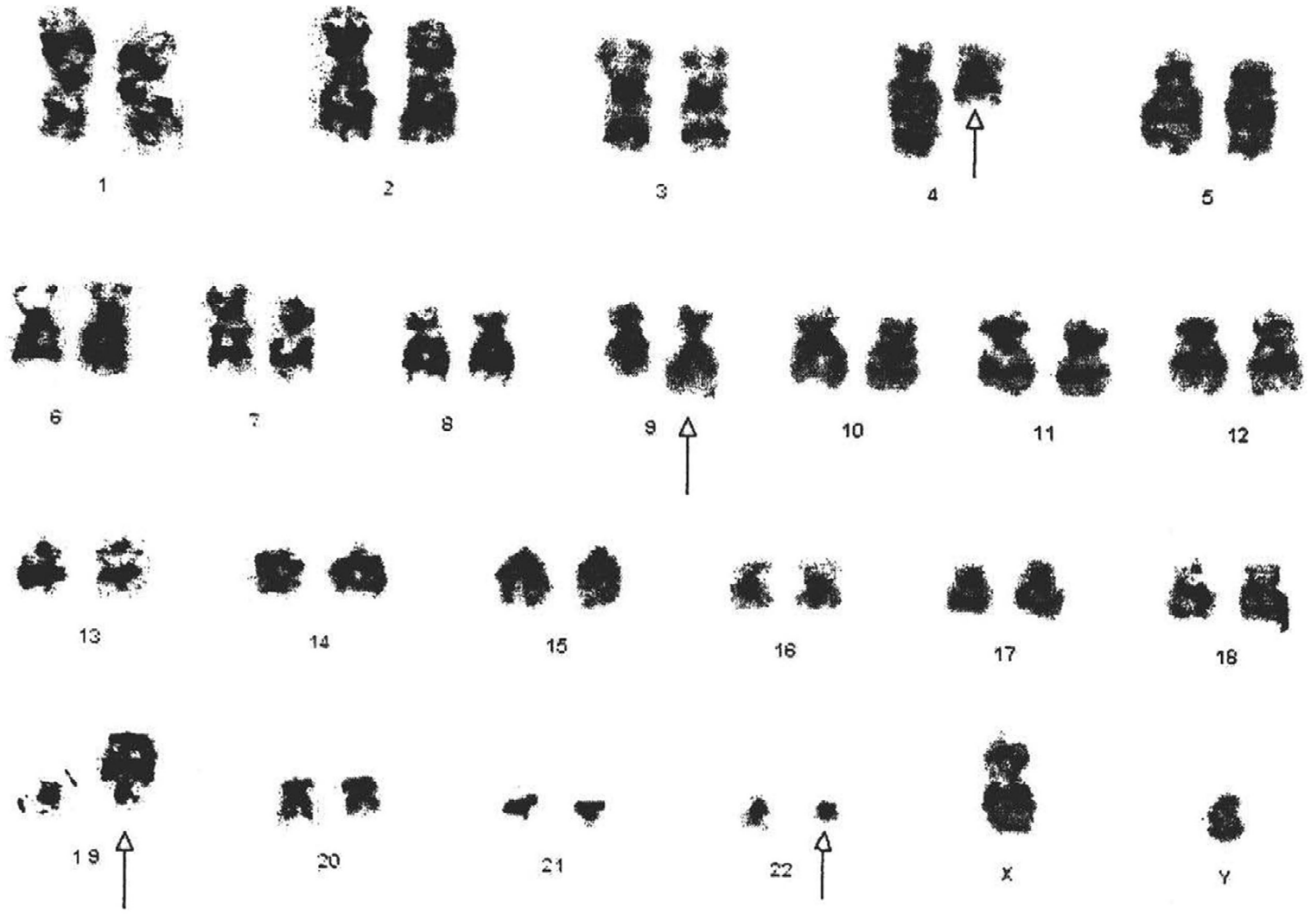
**Cytogenetic analysis shows the karyotype of the CML patient with 46,XY,t(4;9;19;22)(q25:q34;p13.3;q11.2)**. All derivative chromosomes are highlighted by arrow heads.

The cytogenetic analysis showed 46,XY,t(4;9)(q25:q34)t(9;22)(q34;q11.2). Twenty-five cells were counted, and all were positive for Ph chromosome (Figure 1).

**Figure 2:**
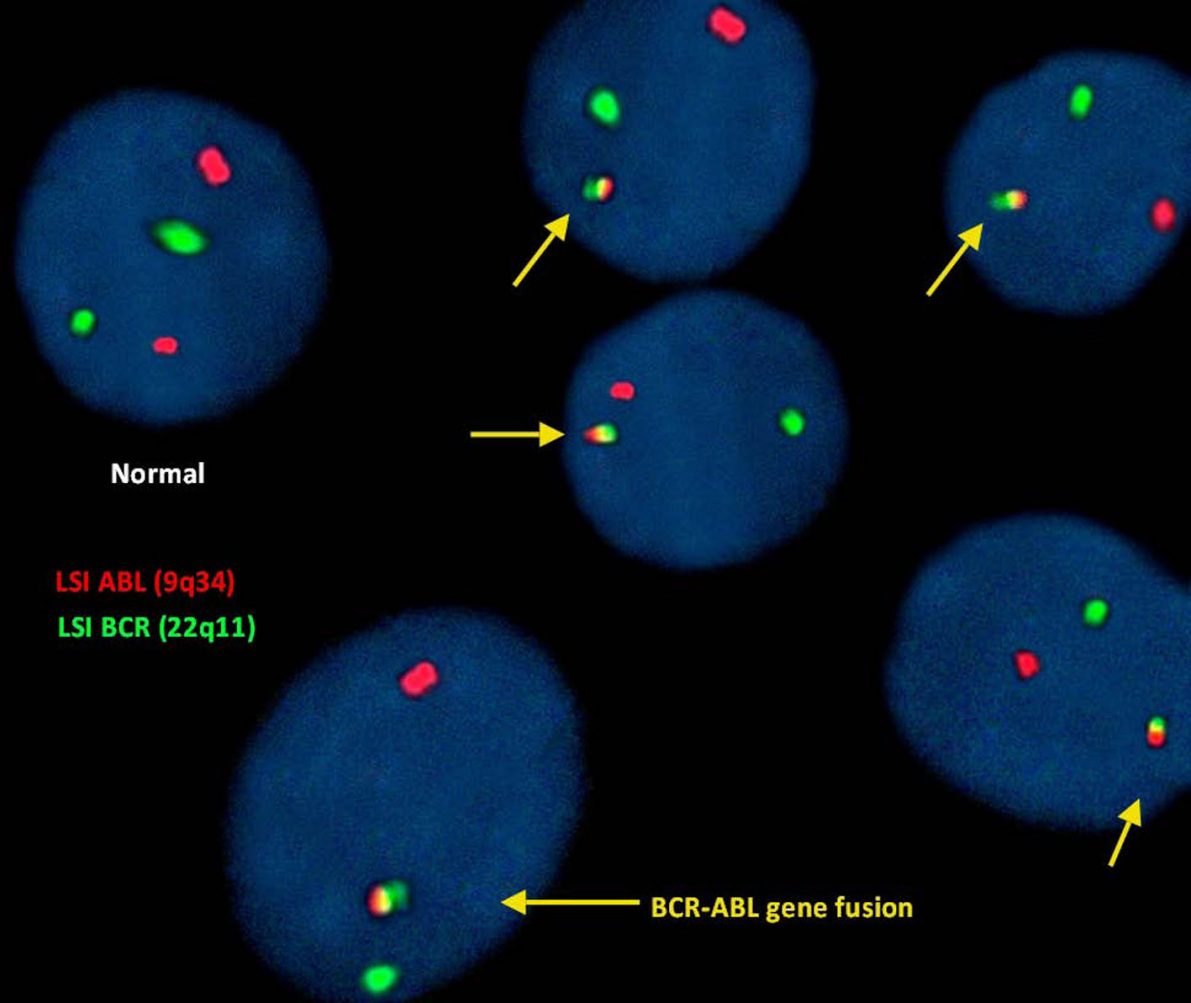
**Fluorescence *in situ* hybridization (FISH) for the detection of (9;22)(q34;q11)**. BCR–ABL translocation was detected in 91% of the 500 nuclei counted.

The BCR–ABL translocation was detected by fluorescence *in situ* hybridization (FISH) analysis in 91% of the 500 nuclei counted. In this study, FISH analysis of the ABL (9q34) gene was identified by fluorescent red dots and BCR (22q11) gene by green dots. Therefore, a cell exhibiting two separate green and red dots counted as a normal cell shows no translocation. However, the irregular translocation in a cell was identified by one red and one green and fused red, yellow, and green signal. Dual color, dual fusion translocation probes were hybridized to patient’s interphase nuclei (Figure 2).

Clinical analysis shows the induced level of WBC (160.7 × 10^9^/L) and low level of Hb (10 mg/dL), which indicates anemia in the CML patient.

The X-ray analysis studies showed no active pulmonary or pleural lesion, normal cardiac and aorta, normal hilar and mediastinal shadows, normal costophrenic angles, normal domes of diaphragm, and normal bony thoracic cage with the normal finding in the chest study. The analysis of liver showed a slight enlargement in size, whereas spleen was massively enlarged, with normal gallbladder, pancreas, kidneys, and retroperitoneum with mild hepatomegaly.

HBsAg and anti-HCV tests were performed by an immunochromatographic screening method, which showed negative results. The patient was treated with antibiotics and Gleevec (imatinib mesylate) 400 mg/day. Socioeconomic status of the CML patient was middle class, and he was aware of this disease.

## Discussion

Chronic myeloid leukemia is primarily caused by the balanced translocation between the long arms of 9;22 chromosomes and secondarily by the variant and complex translocation patients. In such cases, the third, fourth, or even fifth chromosome was involved and is termed as four-, five-, or six-way translocation (6). The four-way translocation is rare; only 59 cases are reported in the literature. The four-way translocation is observed more in male than in female. The five-way translocation is very rare in the CML patients, with only few cases are on record (Table 1).

**Table 1:** **Number of complex variant four-way Ph chromosome translocations reported in the literature**.

Case no.	Karyotype of four-way translocation	Reference
1	46,XX,t(6;8;9;22)(q25;q22;q34;q11)	(13)
2	46,XX,t(2;8;9;22)(p2?3;q1?3;q34;q11)	(14)
3	46,XY,t(1;1;9;22)(p34;q42;q34;q11)	(15)
4	46,XY,t(9;12;12;22)(q34;q21;p12;q11)	
5	46,XY,t(9;22;7;1)(q34;q11;q22;p13)	(16)
6	46,XY,t(1;7;19;22)(q?;q?;p?;q11)	(17)
7	46,XX,t(6;9;22;11)(p21;q34;q11;q13)	(18)
8	46,XY,t(9;22;20;12)(q34;q11;q12;p13)	(19)
9	46,XX,t(2;9;22;7)(q31;q34;q11;q34)(q31;q34;q11;q34)	(20)
10	46,XX,t(9;22;19;10)(q34;q11;p13;q22)	(21)
11	46,XY,t(9;22;11;12)(q34;q11;q12;p13)	
12	46,XY,t(9;22;16;16)(q34;q11;q12;q22)	
13	46,XX,t(9;22;19;10)(q34;q11;p13;q22)	
14	46,XY,t(3;9;22;13)(p14;q34;q11;p13)	
15	46,XY,t(7;11;9;22)(q22;q23;q34;q11)	
16	46,XY,t(2;9;14;22)(p21;q34;q32;q11)	(22)
17	46,XX,t(1;9;22;19)(q32;q34;q11;p13)	(23)
18	46,XX,t(5;6;9;22)(q35;p12;q34;q11)	
19	46,XY,t(9;22;11;15)(q34;q11;p15;q24)	
20	46,XY,t(1;9;19;22)(q21;q34;p13;q11)	
21	46,XY,t(9;22;17;17)(q34;q11;q25;q12)	
22	46,X?,t(7;8;9;22)(p15;q22;q34;q11)	(24)
23	46,XY,t(9;16;9;22)(p22;p13;q34;q11)	(25)
24	46,XX,t(8;12;9;22)(p21;q21;q34;q11)	(26)
25	46,X?,t(7;9;11;22)(p11;q34;q22;q11)	(27)
26	46,XX,t(2;9;13;22)(q33;q34;q32;q11)	
27	46, XX t(9;22;19;10)(q34;q11;p13;q22)	(28)
28	46,XY,t(1;3;9;22)(p12;q22;q34;q11)	(29)
29	46,XY,t(6;6;9;22)(q21;p21;q34;q11)	(30)
30	46,XY,t(6;13;9;22)(p21;q32;q34;q11)	(31)
31	46,XX,t(2;9;22;20)(q37;q34;q11;q12)	(32)
32	46,XX,t(9;22;17;11)(q34;q11;p13;q13)	(33)
33	47,XX,t(3;9;22;12)(q12;q34;q11;p13)	(34)
34	46,XY,t(9;22;16;17)(q34;q11;?;?)	(35)
35	46,XY,t(5;9;22;17)(q12;q34;q11;q11)	
36	46,XY,t(1;19;9;22)(p36;q13;q34;q11)	(36)
37	46,XY,t(3;9;22;12)(p21;q34;q11;q22)	(37)
38	46,XY,t(3;12;9;22)(p21;p13;q34;q11)	(38)
39	46,XY,t(9;22;9;11)(q34;q11;p22;q23)	(39)
40	46,XY,t(6;12;9;22)(p21;q24;q34;q11)	(40)
41	46,XY,t(9;22;15;21)(q34;q11;q15;q11)	(41)
42	46,XY,t(5;7;9;22)(q13;q11;q34;q11)	(42)
43	46,XX,t(5;9;22;17)(q33;q34;q11;p12)	
44	46,XX,t(6;9;22;8)(q24;q34;q11;q24)	
45	46,XY,t(9;22;16;19)(q34;q11;p12;q13)	
46	46,XX,t(8;9;22;10)(q22;q34;q11;p13)	(43)
47	46,XY,t(9;22;14;15)(q34;q11;p11;q21)	
48	46,XY,t(2;9;22;19)(q24;q34;q11;q14)	
49	46,XY,t(4;9;22;21)(q12;q34;q11;q22)	
50	46,XX,t(4;7;9;22)(q21;q15;q34;q11)	
51	46,XX,t(6;9;22;21)(p11;q34;q11;q22)	
52	45,X,-Y,t(8;9;22;15)(q24;q34;q11;q25)	
53	46,XY,t(9;22;12;13)(q34;q11;q11;q13)	
54	46,XX,t(8;22;10;9)(q22;q11;q22;q34)	(7)
55	46,XY,t(9;11;21;22)	(44)
56	46,XY,t(1;9;22;7)(p36;q34;q11;q32)	(45)
57	46,XX,t(9;22;14;20)(q34;q11;q32;q11)	(46)
58	46,XX,t(9;22;19;20)(q34;q11;p13;q22)	(47)
59	46,XX,t(6;9;12;22)(p22;q34;q13;q11)	(48)

**Figure 3:**
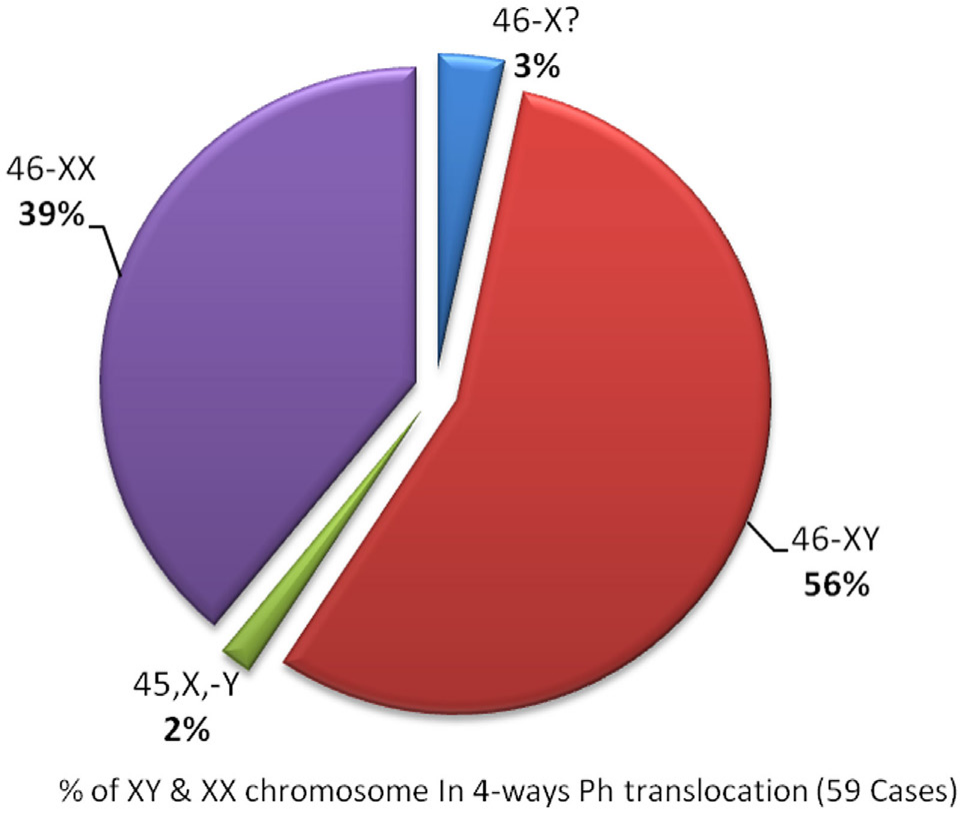
**Percentage of four-way complex variant translocation in male and female CML patients**.

In this study, we reported this new four-way translocation 46,XY,t(4;9;19;22)(q25:q34;p13.3;q11.2) in a CML patient for the first time and was cross-checked in Mitelman Database of Chromosome Aberrations and Gene Fusions in Cancer. In four-way Ph chromosome translocation, male patients are 46-XY,t(56%) who are more affected compared to female patients who are 46-XX,t(39%) (Figure 3), whereas in addition to chromosome 9;22, chromosome 4 (2%) is least compared to chromosome 19, 12, 6 (9%) in four-way Ph chromosome translocation (Figure 3). BCR–ABL translocation was detected in 91% of the 500 nuclei counted. Dual color, dual fusion translocation probes were hybridized to patient’s interphase nuclei. Normal nuclei lacking the t(9;22) translocation showed two green and two orange signals. In the nucleus containing a simple balanced t(9;22), one green and one orange signal from the normal 9 and 22 chromosomes and two green/orange (yellow) fusion signals, one each from the chromosomes 9 and 22.

Mkrtchyan et al. have described two sustainable mechanisms for the formation of variant complex translocation, namely, a single incident rearrangement *via* the simultaneous breakage of few chromosomes followed by mismatched joining and a multistep process of classical Ph translocation followed by additional translocations in chromosomes 9 and 22, as well as other chromosomes (49).

Imatinib mesylate (Gleevec) potentially inhibits BCR–ABL protein tyrosine kinase. Furthermore, it inhibits the tyrosine kinase activities of the platelet-derived growth factor (PDGF) receptor β and c-Kit, but it does not inhibit Flt-3 and Fms belonging to type III tyrosine kinase family (50, 51). Being a first-line oral therapy, imatinib mesylate, an inhibitor of tyrosine kinase activity of BCR–ABL protein, is recommended for Ph-positive chromosome-associated abnormalities. Hence, it would be useful in three-way, four-way, and five-way complex variant translocations, as reported earlier by other research groups (6, 21).

Finally, a unique case of four-way complex variant Ph-positive translocation involving chromosome 46,XY,t(4;9;19;22)(q25:q34;p13.3;q11.2) in a CML patient was reported in this study.

## Author Contributions

MA, AH, MR, and PP: concept design execution. MA, AM, and AH: experimental lab work, execution. SK, MM, AA, and SI: data collection. MR, MJ, AK, MN, HC, KK, AI, ZA, and PP: data analysis and writing.

## Conflict of Interest Statement

The authors declare that the research was conducted in the absence of any commercial or financial relationships that could be construed as a potential conflict of interest.
